# Transactions of Odontological Society of Pennsylvania

**Published:** 1885-01

**Authors:** 


TRANSACTIONS OF THE ODONTOLOGICAL SOCIETY OF PENNSYLVANIA.—From its organization to the close of the year 1883. This is a valuable record of the proceedings of one of the best dental societies in this country and contains much interesting and important information, embraced in over two hundred pages with a number of illustrations. Copies can be obtained from the secretary Dr. Ambler Tees, 548 N. 17th St., Philadelphia.

DIAGNOSIS AND TREATMENT OF CHRONIC NASAL CATARRH.—By Geo. Morewood Lefferts, A. M. M. D., Professor in New York College of Physicians and Surgeons, etc., etc. Reprinted from *Medical News*, and from American Clinical Lectures. Publishers: Lambert & Co., St. Louis, Mo., 1884. A neat little volume of forty-nine pages, in which the subject is scientifically treated.

VICK'S FLORAL GUIDE, 1885.—Published by James Vick, Rochester, N. Y. This is another of the beautiful Annuals published by one of the most celebrated florists and horticulturists in this country, whose seeds and plants have for many years been presented to the public with the most satisfactory results. The present volume is embellished with a beautiful colored frontispiece representing Asters, besides being profusely illustrated throughout. Valuable information can be obtained by a perusal of its pages.

